# Ototoxicity of artemether/lumefantrine in the treatment of falciparum malaria: a randomized trial

**DOI:** 10.1186/1475-2875-7-179

**Published:** 2008-09-16

**Authors:** Robert Gürkov, Teferi Eshetu, Isabel Barreto Miranda, Nicole Berens-Riha, Yoseph Mamo, Tsinuel Girma, Eike Krause, Michael Schmidt, John-Martin Hempel, Thomas Löscher

**Affiliations:** 1 Department of Otorhinolaryngology Head and Neck Surgery, Ludwig Maximilians University, Munich, Germany; 2 Department of Microbiology, Parasitology and Immunology, Jimma University, Jimma, Ethiopia; 3 Department of Infectious Diseases and Tropical Medicine, Ludwig Maximilians University, Munich, Germany; 4 Department of Internal Medicine, Jimma University, Jimma, Ethiopia; 5 Department of Paediatrics, Jimma University, Jimma, Ethiopia; 6 Department of Medical Informatics, Biometrics, and Epidemiology, Ludwig Maximilians University, Munich, Germany

## Abstract

**Background:**

Due to increasing drug resistance, artemisinin-based combination chemotherapy (ACT) has become the first-line treatment of falciparum malaria in many endemic countries. However, irreversible ototoxicity associated with artemether/lumefantrine (AL) has been reported recently and suggested to be a serious limitation in the use of ACT. The aim of the study was to compare ototoxicity, tolerability, and efficacy of ACT with that of quinine and atovaquone/proguanil in the treatment of uncomplicated falciparum malaria.

**Methods:**

Ninety-seven patients in south-west Ethiopia with slide-confirmed malaria were randomly assigned to receive either artemether/lumefantrine or quinine or atovaquone/proguanil and followed-up for 90 days. Comprehensive audiovestibular testing by pure tone audiometry (PTA), transitory evoked (TE) and distortion product (DP) otoacoustic emissions (OAE) and brain stem evoked response audiometry (BERA) was done before enrolment and after seven, 28 and 90 days.

**Results:**

PTA and DP-OAE levels revealed transient significant cochlear hearing loss in patients treated with quinine but not in those treated with artemether/lumefantrine or atovaquone/proguanil. TE-OAE could be elicited in all examinations, except for three patients in the Q group on day 7, who suffered a transient hearing loss greater than 30 dB. There was no evidence of drug-induced brain stem lesions by BERA measurements.

**Conclusion:**

There was no detrimental effect of a standard oral regimen of artemether/lumefantrine on peripheral hearing or brainstem auditory pathways in patients with uncomplicated falciparum malaria. In contrast, transient hearing loss is common after quinine therapy and due to temporary outer hair cell dysfunction.

## Background

Resistance to antimalarial drugs is a common challenge in malaria endemic areas worldwide. Due to increasing resistance of *Plasmodium falciparum *strains against chloroquine and sulphadoxine/pyrimethamine, several Asian and African countries have changed their national policy towards first-line treatment with artemisinin-based combination chemotherapies (ACT) as recommended by current WHO guidelines [1,2].

Some case reports [3,4] as well as a recent alarming report on the possibility of irreversible ototoxicity of artemether/lumefantrine in a retrospective evaluation of construction site workers in Mozambique [5] have raised concerns that this potentially serious side effect of ACT has not been addressed thoroughly enough, although no evidence of neurological side effects or ototoxicity has been observed in human safety studies or large-scale field trials [6] and case control studies [7-9].

Ototoxicity has been reported in association with the use of quinoline type antimalarials [10,11], and quinine since long is known to cause reversible hearing loss and tinnitus[12]. Ototoxic effects have not been reported with the use of some other antimalarials in current use, such as atovaquone/proguanil.

To clarify the question of artemisinin-induced hearing loss, this study integrated a comprehensive neuro-otologic assessment into an investigator-initiated, open-label, randomized, controlled study to compare artemether/lumefantrine with quinine and atovaquone/proguanil in the treatment of uncomplicated falciparum malaria.

## Methods

### Study area and population

The study was carried out at Jimma University (JU) Hospital in the city of Jimma, 1,700 m above sea level and 335 km south west of Addis Ababa, Ethiopia. In this region, malaria transmission is seasonal with peaks from April to June and from September to December during and after the rainy seasons [13]. The first-line treatment of uncomplicated falciparum malaria changed from sulphadoxine/pyrimethamine to AL in Ethiopia in 2004 [14]. Since the drug has not yet been available until recently, oral quinine has mainly been used instead.

Patients over five years of age and suitable for complete audiovestibular testing with parasitologically proven uncomplicated falciparum malaria were recruited from April until August 2006. Uncomplicated falciparum malaria was defined as asexual parasitaemia of less than 100,000/μl blood, acute fever or a history of fever within the preceding 24 hours, and no signs or symptoms suggesting complicated or severe malaria as defined by WHO [15]. Patients with significant hearing loss as determined by failure to detect transitory evoked otoacoustic emissions in either ear, intake of anti-malarial treatment within the previous seven days, severe underlying conditions or concomitant disease masking assessment of response, history of allergy or intolerance against study medications, or pregnancy were excluded. Audiometric testing (duration 1–1.5 h) of eligible patients at enrolment was completed before starting antimalarial treatment.

The study was approved by the Ethical Committee of Jimma University, Ethiopia. Written informed consent was obtained from each patient or the parental guide. Personal subject data are kept confidential. The trial is registered with ClinicalTrials.gov, number NCT00451139. The recommendations guiding physicians in biomedical research involving human subjects issued by the World Medical Association Declaration of Helsinki (Edinburgh, 2000) were applied to this project.

### Antimalarial drug regimens

Eligible patients were consecutively stratified according to gender and age and assigned to one of the following treatment groups by stratified random sampling:

1. Artemether/lumefantrine (AL): 20 mg of artemether and 120 mg of lumefantrine (children 5–14 kg bwt.) or 40 mg/240 mg (children 15–24 kg bwt.) or 60 mg/360 mg (children 25–34 kg bwt.) or 80 mg/480 mg (adults and children ≥35 kg bwt.), at hrs. 0, 8, 24, 36, 48 and 60 (6 doses). Patients were instructed to take the doses with high fat food.

2. Quinine sulphate (Q): 10 mg/kg (children) or 600 mg (adults and children ≥50 kg bwt.), according to about 8 mg/kg Quinine base, three times daily for 7 days (21 doses).

3. Atovaquone/proguanil (AP): 20 mg/8 mg/kg (children < 40 kg bwt.) or 1000 mg/400 mg (adults and children ≥40 kg bwt.) per day for 3 days (3 doses).

Artemether/lumefantrine 20/120 mg tablets (Coartem^®^, Novartis, manifactured by Bejing Novartis Pharma Ltd, Bejing China) and quinine sulphate 300 mg tablets (Remedica Ltd, Limassol-Cyprus-Europe) were obtained from the Ethiopian governmental drug programme through the JU hospital pharmacy. Blister packs of atovaquone/proguanil (Malarone^®^) were purchased from GSK, Germany.

### Procedures

Patients were treated on an outpatient basis and returned on day 7, 28 and 90 and on any day during the follow-up period if symptoms returned. Clinical reassessments covered assessments for potential treatment failure and for potential adverse reactions to the treatment drug including complete audiovestibular testing.

Finger prick blood samples were taken at day 0 for confirmation of *P. falciparum *mono-infection and calculation of parasitaemia (parasites per 200 white blood cells, calculation based on an assumed mean WBC count of 8,000/ml) by microscopic assessment of Giemsa-stained thick and thin blood smears. Aliquots of 10 μl of capillary blood were spotted to Whatman 3 MM Chr filter paper, air dried, and stored at ambient temperature for later molecular analysis. Follow-up blood samples were obtained at days 7 and 28 as well as on any day of potential clinical treatment failure. Patients with falciparum or vivax malaria during the follow-up period were treated according to national guidelines [14]. These patients were excluded from the study after treatment. Patients who completed examinations on day 28 were included in the audiovestibular analysis.

Audiovestibular tests were performed by an examiner blinded to treatment allocation in a separate building specifically designated for this purpose. Although a sound-proof chamber was not available, care was taken to reduce ambient noise to a minimum (see below). Clinical audiovestibular evaluation at each visit included the history of specific complaints (i.e., hearing loss, otalgia, tinnitus, vertigo), otoscopy, Weber and Rinne tests, examination for spontaneous and head-shaking nystagmus under Frenzel glasses, and testing the vestibular function by rapid passive head rotation. Physical examination included gait, Romberg and Unterberger test, finger-to-finger test, and hand rapid alternating movements [16].

Pure tone audiometry was performed with a Madsen Midimate 622D diagnostic audiometer (GN Otometrics, Copenhagen, Denmark) and Beyer DT 48 headphones. The thresholds for frequencies from 125 to 8000 Hz were determined via air conduction. In addition, bone conduction thresholds for 250 to 6000 Hz were determined in order to exclude conductive hearing loss.

In contrast to conventional audiometry, the detection of otoacoustic emissions does not rely on the patients cooperation, and is an excellent indicator for physiologic inner ear function. Transitory evoked (TE) and distortion product (DP) otoacoustic emissions (OAE) were measured with a Cochlea Scan^® ^device (Fischer Zoth, Germering, Germany). TEOAE were analysed using a screening protocol giving a pass (detectable TEOAE) vs. fail result. DPOAE levels were measured at frequencies of f_2 _= 1.5, 2, 3, 4, 6 kHz with at least three different primary tone levels per frequency between 15 and 65 dB (f_2_/f_1 _= 1.2). From the resulting DPOAE growth functions, DP thresholds and estimated hearing thresholds are calculated based on normative data [17,18]. The average noise floor during measurements was -1.2/-2.6/-4.1/-3.6/-3.5 dB for 1.5/2/3/4/6 kHz.

Brain stem evoked response audiometry (BERA) examines the velocity of nerve signal conduction along the auditory pathways from the cochlea to the brainstem, and is the gold standard for detection of damage to the participating neural structures. The measurements were done using an evoselect system (Pilot Blankenfelde, Blankenfelde, Germany). The stimulus, a click of alternating polarity, was delivered at a rate of 11.1 Hz at a level of 80 dB HL and contralateral masking at 40 dB HL to patients resting in a supine position. 2000 measurements were averaged and the absolute and interpeak latencies of Jewett waves I, III and V determined.

For molecular typing sample DNA was extracted from filter paper bloodspots using Chelexò (Bio-Rad, Germany) as described elsewhere [19]. Parasite species was confirmed by nested polymerase chain reaction (PCR) [20]. Sequences of parasite genes coding for the polymorphic merozoite surface proteins (MSP) 1 and 2 were amplified by nested PCR and analysed by restriction fragment length polymorphisms (RFLP) technique [21,22]. MSP1 and MSP2 fragment patterns of isolates from patients with parasite re-appearance were compared with those of the respective recruitment isolates to distinguish recrudescences from new infections [23].

### Statistical analysis

A sample size of 23 in each group was calculated to have 90% power to detect a difference in means of 5.0 dB (e.g., the difference between a group 1 mean of 5.0 and a group 2 mean of 0.0) assuming that the common standard deviation is 5.0 dB using a two group t-test with a 0.05 two-sided significance level. In order to avoid problems with the assumption of normal distribution and to compensate for possible drop-outs it was decided to increase the sample size to at least 30 per group. Data were analysed using SSPS 14.0 software for descriptive statistical analyses and R package V2.4.0 for multivariate methods. The baseline characteristics of the patients were compared by Kruskal-Wallis test for variables that are measured on a continuous scale or by Pearson Chi-square test for categorial variables. The audiological data in a longitudinal setting were fitted in mixed linear model approaches. Potential factors included in the models were day after enrolment, therapy group and side. Variables not significant on the 5% level were sequentially eliminated from the models with exception of the therapy group. In case measurements of the left and right side showed no significant differences, both values were treated in the models as repeating measurements. Generally, a p value of < 0.05 was considered significant.

## Results

**Figure 1:**
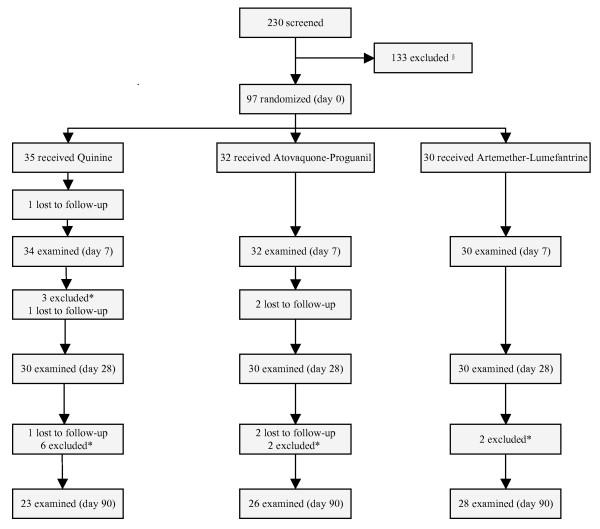
**patient flow chart**. Enrollment, randomization and follow-up of the patients. § The reasons for exclusion (number of patients) were ear discharge (20), impacted ear wax (18), repeated previous otitis media (14), perforated ear drum (6), negative TEOAE recording (15), Weber test lateralized (10), self-treatment with chloroquine (18), mixed infection (17), pregnancy (15). * Patients treated with a second course of antimalarials because of recrudescence or new infection were excluded from audiovestibular evaluation.

230 patients with suspected malaria were screened and 133 were excluded for various reasons (Figure 1). 97 patients were included and randomized. 30 patients received AL, 35 Q, and 32 AP (Figure 1). Baseline characteristics are given in Table 1. None of the patients had received an artemisinin compound before. Cumulated numbers of patients lost to follow-up were one at day 7, four at day 28, and seven at day 90. Thirteen patients were excluded due to recrudescence or new infection (Figure 1).

**Table 1:** Baseline characteristics of the patients who reached the primary end-point (day 28)

		**Quinine**	**Atovaquone/Proguanil**	**Artemether/Lumefantrine**
**n**		30	30	30

**sex**	m/f	16/14	16/14	18/12

**age**	range	6 – 50	6 – 35	8 – 40
	mean	19.2	19.9	18.1
	median	16	18	17

**Temperature (C°)**	range	36.0 – 39.5	36.0 – 39.3	35.6 – 39.9
	mean	37.4	37.3	37.5
	median	37.2	37.3	37.4

**Parasitaemia**	range	360 – 63,000	400 – 69,880	480 – 83,600
	mean	15,469	13,028	21,189
	median	6,740	4,960	9,300

**Symptoms**	Headache	30	30	30
	Nausea/vomiting	21	17	22
	Shivering	23	26	21
	Diarrhea	4	2	3

**Actual daily dose (mg/kg)**	range	30.5 – 36	15 – 23	2.3 – 4.6
	mean	34.9	19.5	3.4
	stdev	1.6	1.9	0.6

Baseline patients' characteristics of the patients who reached the primary end-point (day 28) were not significantly different between the three treatment groups in respect to age (Kruskal-Wallis-test, p = 0,504), sex (χ^2^-test, p = 0,835), parasitaemia (Kruskal-Wallis-test, p = 0,444), body temperature (Kruskal-Wallis-test, p = 0,860). There is also no significant difference in these baseline characteristics of the treatment groups when comparing all 97 treated patients.

### Clinical and parasitological efficacy

On day 7 no treatment failure was detected in any group (Table 2). Until day 28, three patients in the Q group and one in the AP group presented with falciparum malaria. Another patient with asymptomatic parasitaemia was identified in the AP group. The parasitological failure rate on day 28 was 9% and 6% in the Q and AP group, respectively. There was no treatment failure in the AL group. All treatment failures were recrudescences as confirmed by genotyping.

**Table 2:** Clinical and parasitological efficacy

**PCR-corrected failure rates**	**A/L**	**Quinine**	**A/P**
Clinical failure rate day 7	0/30	0/35	0/32
Parasitological failure rate day 7	0/30	0/35	0/32
Intention to treat failure rate* day 7	0/30	1/35 (~3%)	0/32
Clinical failure rate day 28	0/30	3/35 (~9%)	1/32 (~3%)
Parasitological failure rate day 28	0/30	3/35 (~9%)	2/32 (~6%)
Intention to treat failure rate* day 28	0/30	7/35 (~20%)	4/32 (~13%)

**Recurrent parasitaemia**			

Number of patients with recrudescence** (day)	1 (70)	4 (24, 28, 28, 40***)	2 (28, 28)
Number of patients with new infection** (day)	1 (73)	4 (34, 40, 40***, 65)	1 (80)
Number of patients with ***P. vivax ***infection (day)	2 (28, 28)	5 (22, 25, 27, 28, 28)	2 (28, 28)

**Gametocytaemia**			

Number of patients on day 0	1	3	1
Number of patients on day 7	2	10	16
Number of patients on day 28	0	2	0

* lost to follow-up included

**P. falciparum: Genotyping by PCR and RFLP patterns of the MSP-1 and MSP-2 gene

*** One sample showed two different clones on day 40, one of them corresponded to the clone on day 0.

Between day 28 and day 90 seven patients with falciparum malaria were diagnosed. Five patients had a new infection. A recrudescent and a new strain were found in a patient in the Q group on day 40, and one recrudescence occurred on day 70 in the AL group.

Nine patients (five treated with Q, two with AP, and two with AL) showed *P. vivax *infection during follow-up (Table 2).

### Tolerability and ototoxicity assessment

No vomiting occurred after ingestion of the antimalarial drugs, and no serious adverse events were reported during treatment and follow-up. Most symptoms present at the time of diagnosis resolved until day 7 (Table 3). However, hearing problems and tinnitus were more common on day 7 with nine of thirty patients complaining of hearing problems in the Q group. In seven of these, audiometry and OAE testing confirmed significant hearing loss. Patients reporting subjective hearing impairment in the AL group did not have abnormal hearing test results. In the AP group, only the reported hearing loss by one patient on day 90 corresponded to significantly impaired audiometry and OAE results; in this patient malaria reinfection was diagnosed.

**Table 3:** Symptoms and clinical signs

Drug Group	**Quinine**				**Atovaquone-proguanil**				**Artemether-lumefantrine**			
**n =**	30	30	30	23	30	30	30	26	30	30	30	28
**day**	0	7	28	90	0	7	28	90	0	7	28	90
												
**Hearing Problem**	0	9	1	0	0	1	0	1	1	2	1	0
**Tinnitus**	2	8	3	0	4	6	1	1	6	4	2	0
**Vertigo**	6	0	1	0	8	1	0	0	4	3	1	0
**Imbalance**	1	0	0	0	0	0	0	0	0	0	0	0
**Spontaneous nystagmus**	0	0	0	0	0	0	0	0	0	0	0	0
**Provoked nystagmus**	0	0	0	0	0	0	0	0	0	0	0	0
**Pathologic head rotation test**	1	0	0	0	0	0	0	0	1	0	0	0
**Impaired coordination**	0	0	0	0	0	0	0	0	0	0	0	0
**Fever**	24	0	5	2	23	2	3	1	26	0	1	2
**Shivering**	23	0	1	2	26	4	3	1	21	1	1	1
**Headache**	30	5	5	2	30	10	6	3	30	7	4	2
**Nausea**	21	1	2	0	17	1	1	0	22	0	0	4
**Diarrhea**	4	0	0	0	2	1	1	0	3	0	0	0
**Vomiting**	1	0	0	0	1	0	0	0	1	0	0	0
**Anorexia**	2	0	1	0	3	0	0	0	0	0	0	0
**Abdominal pain**	1	0	0	0	0	0	0	0	1	0	0	0
**Arthralgia**	3	0	0	0	3	0	0	0	1	0	0	0
**Myalgia**	1	0	0	0	0	0	0	0	0	0	0	0
**Chest pain**	0	0	0	0	0	1	0	0	0	0	0	0
**Cough**	0	1	0	0	0	1	0	0	0	0	0	0

#### Pure tone audiometry

**Figure 2:**
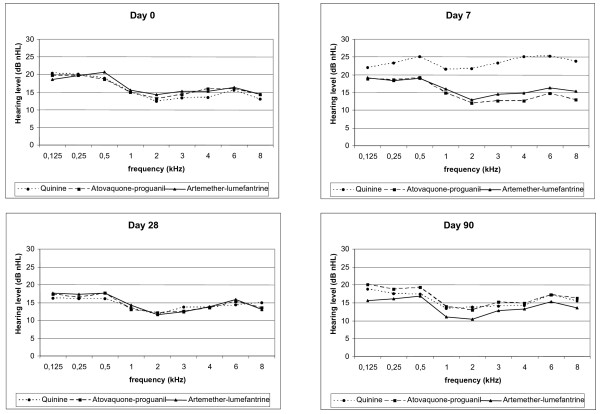
**pure tone hearing levels**. Audiometrically determined mean hearing levels on day 0, 7, 28 and 90. Transient hearing loss in the quinine treated group is observed on day 7. No permanent hearing loss in either group occurred. Measuring unit of y-axis is dB nHL.

Air conduction hearing thresholds were compared for a standard range of frequencies from 0.125 to 8 kHz. Bone conduction thresholds were also measured at all time points in order to exclude a possible conductive hearing loss. Figure 2 shows similar mean hearing levels at day 0 (baseline) for all groups and all frequencies. In the Q group, a hearing loss affecting all frequencies is evident on day 7 and has disappeared by day 28. Otherwise, no significant changes of the mean hearing thresholds compared to day 0 were evident, except for some slight general improvement in all groups.

**Figure 3:**
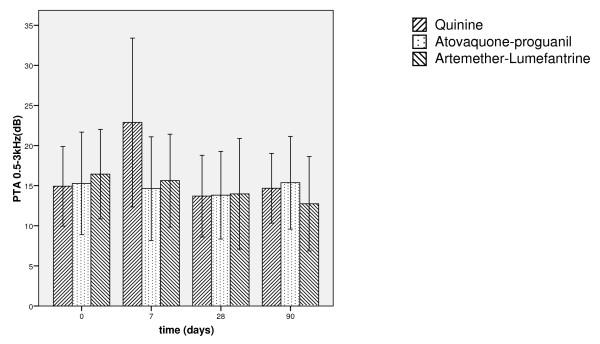
**Pure tone average 0,5–3 kHz**. Plot of means +/- 1 standard deviation of the pure tone average at 0.5, 1, 2, and 3 kHz for day 0, 7, 28 and 90 stratified by therapy group (solid line: Quinine, dashed line: Atovaquone/proguanil, dotted line: Artemether/lumefantrine). As depicted in the graph a slight improvement of PTA was found during the first 28 days after treatment in the Atovaquone/proguanil and Artemether/lumefantrine group unlike the Quinine group, which experienced a marked but transient hearing loss measured on day 7. On day 90, the differences of means were larger than those on day 28, which can be explained as an effect of the long-term observation. No permanent hearing loss in either group occurred. The results presented in the graph are strongly confirmed by multivariate analysis. Measuring unit of y-axis is dB nHL.

When comparing the mean 4-tone-average (0.5, 1, 2, 3 kHz) as the clinically most significant frequency range, a similar picture emerges (Figure 3). Multivariate analysis of the 4-tone-average revealed a strong interaction between the factors group and time on day 7, confirming the temporary threshold shift caused by quinine. Multivariate analysis of the mean average of higher frequencies (4, 6 and 8 kHz) reveals the same effect.

According to the ASHA criteria for ototoxicity [24], there was no evidence of persistent hearing loss in any treatment group.

#### Otoacoustic emissions

**Figure 4:**
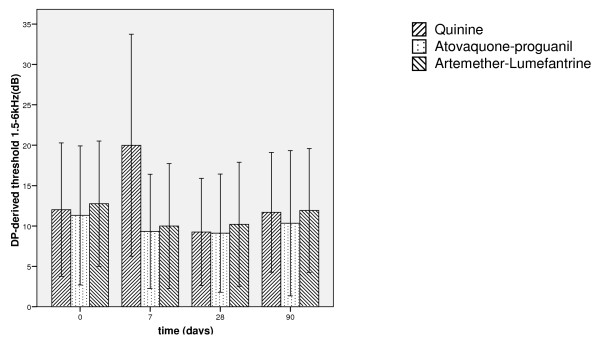
**DPOAE level 1,5–6 kHz**. Mean DPOAE estimated hearing levels +/- 1 standard deviation at 1.5, 2, 3, 4 and 6 kHz (solid line: Quinine, dashed line: Atovaquone/proguanil, dotted line: Artemether/lumefantrine). Estimated hearing levels are elevated in the quinine treated group on day 7. No permanent elevation in estimated hearing levels in either group occurred, which is confirmed by multivariate analysis. Measuring unit of y-axis is dB nHL.

The average DP threshold level of the Q group on day 7 is markedly elevated from baseline, corroborating the pure tone audiometry data (Figure 4). Multivariate analysis reveals a significant effect of time on the DP threshold levels for day 7 and day 28. This is reflected in the general improvement of DP thresholds for these time points when compared to baseline. The three treatment groups do not behave differently, except on day 7 when a significant combined effect of time and group is visible as the Q ototoxicity.

Transitory evoked otoacoustic emissions (TEOAE) could be elicited on both ears in all examinations, except for three patients in the Q group on day 7, in whom TEOAE could not be detected in either ear, and who suffered a transient hearing loss greater than 30 dB.

#### Brainstem evoked response audiometry

**Figure 5:**
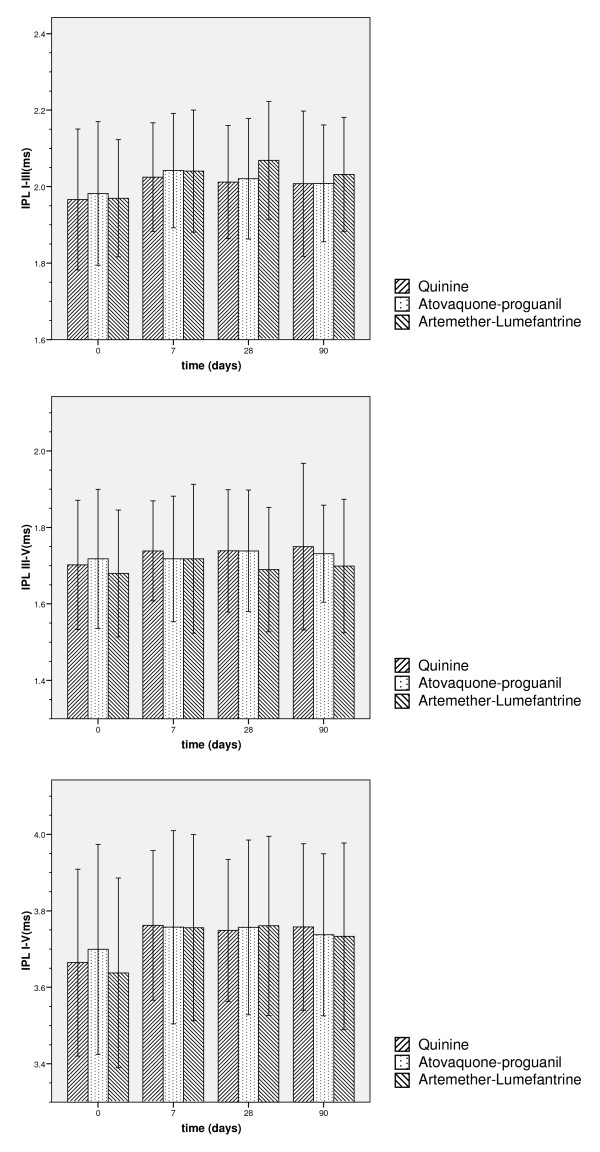
**Interpeak latencies I–V, I–III, III–V**. Mean interpeak latencies between Jewett waves I, III and V +/- 1 standard deviation (solid line: Quinine, dashed line: Atovaquone/proguanil, dotted line: Artemether/lumefantrine). Interpeak latencies in all groups are shorter on day 0, when patients have elevated body temperature. The difference in IPL I–III between the Artemether/lumefantrine group and the other two groups is limited to the right ear. No permanent drug-related prolongation of interpeak latencies occurs, as confirmed by multivariate analysis.

Interpeak latencies (IPL) were calculated for the I–V, I–III and III–V intervals. In all groups, IPL I–V were shorter on day 0 than on later time points (Figure 5). The difference in IPL I–III between the AL group and the other two groups on day 28 was limited to the right ear. However, only one patient in the AL group had a potentially clinically relevant interaural difference of IPL I–III greater than 10% on day 28, which disappeared by day 90. No permanent drug-related prolongation of interpeak latencies occurred.

By comparison of these measurements with normative data (2.49 ms for IPL I–III, 2.16 ms for IPL III–V, 4.45 ms for IPL I–V; [25] ), IPL III–V was prolonged in one Q treated patient (left ear) on day 28, but not on day 90.

## Discussion

The controversy about artemisinins and ototoxicity in humans has only recently been investigated by Toovey *et a*l [5]. The authors compared audiometric data from 150 adult construction site employees who have been treated with AL for uncomplicated malaria with 150 matched controls who neither suffered malaria nor received artemether. Significant hearing loss over the term of their employment was found in frequencies between 1 and 8 kHz. This was judged to be irreversible, because the time between treatment and exit audiogram (mean = 163 days, range 3–392 days) did not correlate with the degree of hearing loss [26]. However, possible confounding factors like the influence of noise exposure in these construction site workers or the lack of a control group of malaria patients treated with other antimalarials, make it difficult to establish a causal relationship between hearing loss and AL therapy from this retrospective evaluation.

Animal studies demonstrated that parenteral admininistration of lipophilic artemisinin derivatives – such as artemether – can induce focal brainstem lesions including auditory and vestibular pathways (reviewed in [27] ). Oral preparations, however, have different pharmacokinetics and do not achieve as high plasma concentrations [28], suggesting that the prolonged presence of artemisinin upon slow release from oil-based intramuscular formulations and the relatively high doses used in animal studies are the main cause of neurotoxicity in laboratory animals.

A post-mortem study examined brains of patients who had died of severe malaria and had received either intramuscular artemether (n = 6) or intravenous quinine (n = 15) in doses exceeding currently deployed regimens and found no evidence of the typical artemisinin lesions observed in animal studies [29]. However, since median time from admission to death in the artemether group was only 76.5 h, delayed neurotoxicity may not have been detected, as suggested from in vitro studies [30].

Detailed neurological data are provided by Price *et al*: of more than 1,000 patients above five years of age treated with artemether or artesunate (alone or in combination with mefloquine) and examined on days 2, 7 and 28 post-treatment, no patient developed deafness (assessed by tuning-fork test) or permanent neurological abnormalities.

A retrospective study carried out in Vietnam [8] compared 337 subjects who had received from two to 21 courses (median = 2) of either artemisinin or artesunate with 108 controls from the same village. Even though 20% of the subjects had received cumulative doses of ≥ 500 mg/kg artemisinin (or the adjusted equivalent of artesunate), which might be more than in any other group of people in the world, the authors found no evidence of a drug effect on screening audiometry (testing for hearing loss ≥ 40 dB), brainstem evoked auditory potential latencies or neurological examination. Similar results were obtained in two case-control studies from Thailand in 79 subjects treated at least twice with oral artesunate or artemether [9] and in 68 patients who had been treated with AL [7].

A recent study in 15 adult volunteers with experimental falciparum malaria treated with AL could not detect any ototoxicity by using conventional and evoked response audiometry, but did not compare artemisinins to other antimalarials [31].

This study is the first randomized clinical trial directly comparing ototoxicity of AL with other antimalarial drugs. Quinine has since long been known to cause hearing loss and tinnitus (cinchonism). Generally, this side-effect is reported to be reversible within about 24 hours [32], although some case reports have described permanent hearing impairment associated with quinine treatment. In guinea pigs given large doses of quinine, there is degeneration of the organ of Corti which begins with loss of the external hair cells, and may further affect the stria vascularis and inner hair cells.

The Q and AP group can be viewed as a "positive control" and "negative control", respectively. Furthermore, an attempt was made to evaluate the study population's audiovestibular systems as comprehensively as possible, including air and bone conduction audiometry for definite exclusion of conductive hearing loss and otoacoustic emissions for direct objective assessment of cochlear function. In addition to the assessment on day 28, patients were examined on a late time-point (day 90) to check for reversibility of any potential hearing loss. In consequence, day 90 data could be biased by the fact that not all patients were available for the day 90 examinations (7 in the quinine group, 4 in the AP group and 2 in the AL group). However, a separate analysis including only patients who completed follow-up until day 90 showed results similar to those depicted.

The relatively low numbers of positive clinical findings or complaints related to the audiovestibular system (Table 3) makes comparison across groups difficult. Nevertheless, the transient quinine ototoxicity on day 7 is clearly correlated with an elevated number of patients complaining of hearing problems. When comparing the time course in the overall patient population, there is a relatively high proportion of patients who complain of tinnitus on day 0 and of tinnitus and perceived hearing loss on day 7. The symptom vertigo which is prevalent primarily on day 0 does not seem to be of vestibular origin, since the clinical vestibular function tests are not significantly abnormal in either group. There were no relevant neurological impairments detected. For practical reasons, this study did not incorporate objective vestibular function tests. Rotatory and caloric vestibular tests necessitate a considerable amount of specialized equipment. Vestibular evoked myogenic potentials (VEMP) recording is now emerging as a reliable tool to assess otolith function and could be useful in future studies, since this can be done with a standard BERA equipment. The air conduction hearing thresholds of our patients show no detrimental influence of AL in comparison to the other two study drugs. In contrast, a significant temporary hearing loss was observed in quinine treated patients on day 7. The observed general tendency of improved hearing levels on later time points in comparison to day 0 could be due to either a learning process or a direct transient negative effect of malaria on hearing or a lack of concentration in the acutely ill patients on day 0. The relatively high thresholds in the lower frequencies probably reflect the presence of some ambient noise.

Otoacoustic emissions are sound signals resulting from the mechanical action of outer hair cells in the organ of Corti. By stimulating the cochlea simultaneously with two signals of specific frequencies and sound pressure levels, distortion product otoacoustic emissions at a third specific frequency are elicited and can be measured. By plotting stimulus (input) sound pressure levels versus DP (output) levels, a DP threshold value can be calculated, i.e. the lowest stimulus level producing an outer hair cell response. Since outer hair cell responses are needed for physiological hearing perception at the hearing threshold, this DP threshold correlates very well with the subjective hearing threshold. It, therefore, provides an objective estimate of hearing levels in patients with mild to moderate cochlear hearing loss [17].

Overall, the objective otoacoustic emission data largely parallel the subjective audiometric data. It is therefore demonstrated that the elevated hearing levels in audiometric measurements were due to the known transient cochleotoxic effect of quinine.

DP thresholds of later time points are generally lower than those on day 0 (except for the quinine group on day 7). This cannot be due to a lack of concentration on day 0 nor to a learning curve, since this test directly assesses outer hair cell function without the need for patients' cooperation. The fact that the DP threshold levels are slightly lower than the pure tone audiometer thresholds is probably due to some ambient noise that could not be avoided in lack of a truly sound-proof chamber.

From the pattern of artemisinin-induced focal brainstem lesions in animal studies, it can be expected that an analogous lesion in humans would lead to a prolongation of the interpeak latency of Jewett waves III–V. The observed shorter interpeak latencies on day 0 are associated with elevated body temperature, a correlation previously described [33,34]. There was no permanent drug-related latency prolongation in the patients treated with AL. A prolonged interpeak or absolute latency, compared with normative data, occurred in none of the AL-treated patients.

## Conclusion

In conclusion, this study could not detect any detrimental effect of a standard oral regimen of AL on peripheral hearing or brainstem auditory pathways in patients with uncomplicated falciparum malaria as assessed by pure tone audiometry, otoacoustic emission recording and brainstem evoked response audiometry. In contrast, this study clearly detects the transient quinine induced hearing loss due to temporary outer hair cell dysfunction. These results therefore support the continued use of oral artemisinine based combination therapy for uncomplicated malaria.

## Competing interests

The authors declare that they have no competing interests.

## Authors' contributions

TL initiated and coordinated the study. TE, RG, IBM, YM, and TG participated in undertaking the clinical studies. TE, IBM, and NBR were responsible for microscopy and molecular analysis. RG, EK, and JMH were responsible for the analysis of the data from audiovestibular measurements.  MS was responsible for statistical analysis. All authors participated in the design, analysis, interpretation, and writing up of the research work and read and approved the final manuscript.
